# Transient Laser-Annealing-Induced Mesophase Transitions of Block Copolymer–Resol Thin Films

**DOI:** 10.1021/acspolymersau.1c00040

**Published:** 2021-12-13

**Authors:** Wei Han Tu, Geok Leng Seah, Yun Li, Xinghui Wang, Kwan W. Tan

**Affiliations:** † School of Materials Science and Engineering, Nanyang Technological University, Singapore 639798, Singapore; ‡ College of Physics and Information Engineering, Institute of Micro-Nano Devices and Solar Cells, Fuzhou University, Fujian 350108, China

**Keywords:** Self-assembly, Laser annealing, Block copolymers, Phenolic resols, Structural evolution, Kinetics, Milliseconds, Non-equilibrium

## Abstract

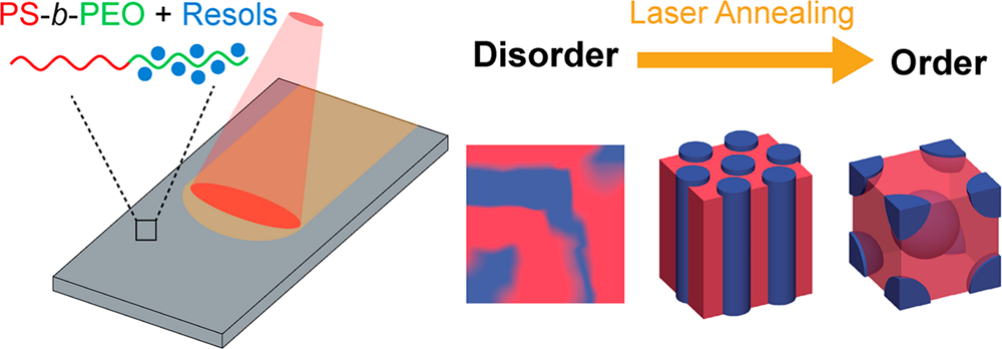

Block copolymer self-assembly-derived thin films provide direct access to two- and three-dimensional periodically ordered mesostructures as enablers for many nanotechnology applications. This report describes laser-annealing-induced disorder–order mesophase transitions of polystyrene-*block*-poly(ethylene oxide)/resol hybrid thin films over a range of laser temperatures (∼45 to 525 °C) and short dwell times (0.25 to 100 ms), revealing the non-equilibrium ordering and disordering kinetics and behaviors. We found that a combination of transient laser temperature of ∼275 °C and annealing dwell time of 100 ms provided the most optimal kinetic and thermodynamic control of the diffusivities of hybrid mesophases and photothermal-induced resol polymerization, yielding long-range ordered films resembling an in-plane body-centered cubic sphere morphology. A clear understanding of hybrid thin film mesophase self-assembly under non-equilibrium laser annealing could open new avenues to introduce novel chemistries and rapidly achieve nanoscale periodic order suitable for the patterning of complex structures, electronics, sensing, and emerging quantum materials.

## Introduction

Amphiphilic block copolymers (BCPs) readily self-assemble into a rich diversity of periodically ordered two- and three-dimensional (2D/3D) mesostructures (5–50 nm) for many nanomaterial applications such as patterning of complex nanostructures, electronics, magnetic storage, energy conversion and storage, separation, and emerging quantum materials.^1−17^ For instance, over the past three decades, there has been intensive research in di-BCPs with high Flory–Huggins interaction parameter, χ, e.g., polystyrene-*block*-polybutadiene,^1^ polystyrene-*block*-poly(methyl methacrylate),^2−5,18−20^ polytrimethylsilylstyrene-*block*-polylactide,^21^ polystyrene-*block*-poly(dimethylsiloxane),^6,22,23^ and polystyrene-*block*-poly(ethylene oxide),^7^ that aims to achieve high density periodic patterns with sub-10 nm features. Recently, zone thermal annealing methods such as laser annealing, microwave annealing, and thermal flash annealing are used to control the self-assembly of neat BCPs to enhance microphase separation kinetics, reduce defects, and attain periodic long-range order.^15−20,22−27^

To increase the functionality and application space, BCPs have been employed as structure-directing agents of organic/inorganic additives to generate organic–organic and organic–inorganic hybrid composites with designer physical and chemical functional properties.^9−15,28−37^ Coupling BCP-directed self-assembly with laser annealing further expands the material property and processing windows beyond traditional limits.^15,38,39^ For example, Tan et al. performed sequential CO_2_ laser irradiation on BCP–resol hybrid films for sub-millisecond dwell times in air, inducing simultaneous photothermal BCP decomposition and resol polymerization to yield 3D mesoporous continuous resin/carbon films with a disordered network morphology (termed as B-WRITE).^10^ The resultant mesoporous resin films were further employed as hard templates for chemical vapor deposition of amorphous silicon (a-Si) into the pore network, followed by nanosecond pulsed excimer laser annealing and template removal, generating complementary 3D mesoporous crystalline silicon (c-Si) semiconductor nanostructures.^9,10,28^ Remarkably, the resol-derived resin template showed excellent thermal and structural resilience above the melting temperature of a-Si (>1250 °C) on such short heating time scales (<10^–5^ s).^10,28^ The BCP–resol nanocasting approach was further adapted by Yu et al. to produce nanosecond excimer laser-induced 3D periodic c-Si nanostructures using a mesoporous gyroidal resin template obtained by solvent annealing.^29,30^ However, conventional thermal and solvent annealing methods to form ordered BCP–resol thin film morphologies typically require long durations up to tens of hours and strict-controlled environments involving organic solvents.^30,35^ To the best of our knowledge, millisecond laser spike annealing (LSA) of the all-organic BCP–resol hybrid films under ambient conditions to obtain periodic mesostructures remains unexplored. Being able to reach high temperatures (>1400 °C) and then rapidly cooled (quench rates up to 10^7^ K/s) on the sub-millisecond and millisecond time scales (0.05–100 ms), LSA can provide thermodynamic and kinetic control of BCP morphological developments by tuning the diffusivities and reaction rates of respective polymer blocks.^20,38^

Here, we report the disorder–order mesophase transitions of polystyrene-*block*-poly(ethylene oxide)/phenol–formaldehyde resol (PS-*b*-PEO/resol) thin films under millisecond LSA. By studying the self-assembly behaviors of the all-organic hybrid films under various laser annealing temperatures (45–525 °C) and annealing durations (0.25–100 ms), two competing photothermal induced kinetic processes toward structure order were elucidated. First, LSA temperatures above the glass transition temperature allowed the hydrophobic PS block to gain increasing mobility to arrange into a more stable ordered microstructure. While the hydrophilic PEO/resol domains also gained increased mobility during laser annealing, simultaneous photothermal induced polymerization of resols into higher molar mass resin eventually led to the loss of mobility, particularly at higher temperatures and very short annealing dwell times. Finally, we obtained the most ordered PS-*b*-PEO/resol hybrid films with an in-plane body-centered cubic sphere morphology after LSA at ∼275 °C for 100 ms dwell time.

## Experimental Section

### Materials

All chemicals were used as received unless described otherwise. The styrene monomer (stabilized with TBC, >99.0%) and *N*,*N*,*N*′,*N*′,*N*″-pentamethyldiethylenetriamine (PMDETA, >99.0%) were obtained from TCI Chemicals. CuBr (99.999%), CuBr_2_ (99.999%), 2-bromoisobutyryl bromide (98%), *p*-toluenesulfonic acid (ACS reagent, ≥98.5%), poly(ethylene glycol)methyl ether (average *M*_n_ ∼ 5000), neutral alumina (A1522, type WN-6, neutral, activity grade super I), polystyrene (PS, average *M*_n_ ∼ 170,000), tetrahydrofuran (THF, anhydrous), anisole (anhydrous), formaldehyde solution (ACS reagent, 37 wt % in H_2_O), and phenol (unstabilized, ≥99%) were obtained from Sigma-Aldrich.

### Synthesis of PS-*b*-PEO/Resol Bulk Monoliths

A linear PS-*b*-PEO with a molar mass of 35.3 kg/mol, a polydispersity index of 1.25, and a composition of 82.5 wt % PS and 17.5 wt % PEO was synthesized by atom transfer radical polymerization (ATRP) as described elsewhere^8^ and characterized by ^1^H NMR spectroscopy and size-exclusion gel permeation chromatography. Oligomeric phenol–formaldehyde resols (<1 kg/mol) in THF (20 wt %) were prepared by the polymerization of phenol and formaldehyde under basic conditions as described elsewhere.^30,34^

First, 0.113 g of PS-*b*-PEO was dissolved in 0.5 g of THF for 30 min; then 0.25 g of resol (20 wt % in THF) was added and stirred for 24 h. The brown-colored transparent solution was cast in polytetrafluoroethylene beakers (5 mL) set on a glass Petri dish covered with a hemispherical glass dome and heated at 50 °C over 24 h for solvent-evaporation-induced self-assembly. The all-organic hybrid monolith samples were then cured in a vacuum oven at 110 °C for 24 h. Mesoporous carbon monolith samples were obtained by pyrolysis in a tube furnace under nitrogen at 600 °C (2 h) with a ramp rate of 1 °C/min.

### Synthesis of PS-*b*-PEO/Resol Thin Films

Germanium/quartz (Ge/quartz) substrates were prepared by sputter deposition of Ge (Ge film thickness of ∼300 nm) on clean quartz substrates (quartz substrate thickness of 1 mm) using a rf magnetron source with argon ions at a base pressure of <5 × 10^–6^ Torr and working pressure of 8 × 10^–3^ Torr (Kurt J. Lesker PVD75). The Ge/quartz substrates were exposed to ambient air plasma for 10 min (Harrick Plasma PDC-32G-2) before hybrid film deposition. PS-*b*-PEO/resol solution was prepared by dissolving 0.028 g of PS-*b*-PEO with 0.06 g of resol (20 wt % in THF) in 1.948 g of THF for 24 h. The ∼120 nm thick all-organic hybrid films were spin-coated on Ge/quartz substrates at 2000 rpm (60 s) in ambient air.

### Millisecond Laser Annealing of Hybrid Films

The transient laser annealing setup is described elsewhere.^10,38,40^ Briefly, a continuous-wave semiconductor laser (wavelength 532 nm, Coherent Verdi G20) focused to a line beam profile full width at half-maximum (fwhm) of ∼100 μm by 400 μm was scanned across the polymer films via dynamic stage motion at velocities of 1 to 400 mm/s, resulting in 400 μm fwhm scan lines for dwell times of 0.25 to 100 ms, all in air. To anneal larger areas, hybrid film samples were scanned by the laser in a raster manner with a 80 μm step size. For the equilibrium thermal annealing experiments, hybrid thin film samples were annealed in a vacuum oven at various temperatures of 100–150 °C for 24 h.

### Characterization

Atomic force microscopy (AFM) images were obtained on an AFM Park Systems NX10 in noncontact mode under ambient conditions. Transmission electron micrographs (TEM) were obtained using a JEOL 2010 electron microscope operating at the accelerating voltage of 200 kV equipped with an AMT XR40B CCD camera. Fast Fourier transform (FFT) analysis was performed on AFM height micrographs in the ImageJ software as described elsewhere.^10^ Scanning electron microscopy (SEM) was conducted using a JEOL 7600F field-emission SEM equipped with a half-in-lens detector to obtain film thicknesses of the sputter-deposited Ge on quartz substrate and the PS-*b*-PEO/resol hybrid films. Profilometry measurements were conducted using a KLA-Tencor Alpha-Step D-500 profilometer. Optical microscopy images were obtained using an Olympus BX53 microscope.

Small-angle X-ray scattering (SAXS) measurements in transmission and grazing incidence modes were collected with a Xenocs Nano-inXider using Cu Kα radiation source and Dectris Pilatus 3 detectors. The 2D SAXS patterns were azimuthally integrated around the beam center into 1D scattering intensity curves plotted against the scattering vector magnitude = 4π sin θ/λ, where θ is half of the total scattering angle and λ is the X-ray wavelength. The *d*-spacing values were calculated using *d* = 2π/q*, where q* is the scattering vector of the principal peak. Differential scanning calorimetry (DSC) measurements were conducted using a TA Instruments DSC Q10 for thermal cycles between −50 and 300 °C with heating/cooling rates of 20 °C/min. Thermogravimetric analysis (TGA) measurements were conducted using a TA Instruments Q500 under nitrogen with a heating rate of 10 °C/min.

### Peak Laser Annealing Temperature Simulations and Absolute Temperature Calibrations

Finite element modeling (FEM) simulations were performed to estimate the peak annealing temperature under laser irradiation.^41^ The 2D simulation model is a Ge/quartz substrate with a 25 mm long by 300 nm thick Ge overlayer on a 25 mm long by 1 mm thick quartz substrate that is placed on top of a 100 mm long by 10 mm thick aluminum block (linear stage). The 532 nm laser heat source, *Q* (see eqs S1 and S2), was modeled as a Gaussian line with beam profile *r*_*x*_ and *r*_*z*_ of 340 and 85 μm, respectively, where *r* = (1.699 × fwhm)/2. It is reasonable to assume the Ge/quartz substrate is thermally isotropic and the Ge overlayer absorbs most of the laser photons to heat the polymer films.^10,18,38^ All surfaces were subjected to surface-to-ambient radiation using eq S3. The simulated peak temperature *T* of the Ge/quartz substrate was computed using eq S4. All material properties^42^ are summarized in Table S1, and the FEM mesh model schematic is shown in Figure S1. We further performed temperature calibrations based on the glass transition and decomposition of ∼200 nm thick films of PS on Ge/quartz substrates heated by a single laser irradiation at 0.01 to 0.50 W for 100 ms dwell time.^18,40^ The peak temperature in the center of the laser scan is dependent on the laser power and heating dwell time.^40^ The heating and cooling rates were obtained from the gradient of tangent lines connecting the peak temperature to the ∼50% peak temperature point in the respective simulated temperature profiles.

## Results and Discussion

### Equilibrium Morphologies of Bulk and Thin Film PS-*b*-PEO/Resol Hybrids

**Figure 1:**
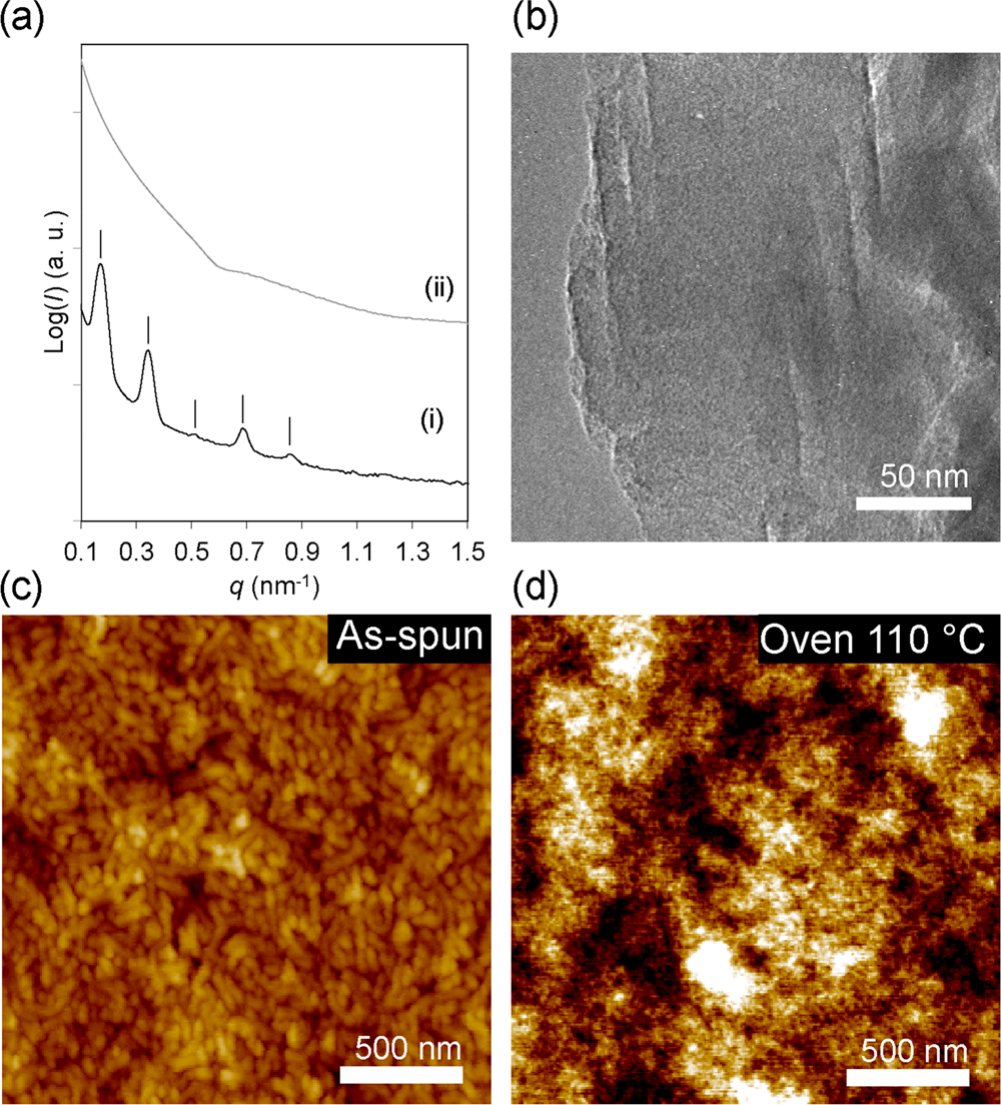
(a) SAXS patterns of bulk monolithic samples of (i) PS-*b*-PEO/resols hybrid and (ii) mesoporous carbon after pyrolysis at 600 °C. Tick marks in (a) indicate the expected reflections for the lamellar structure. (b) TEM of monolithic mesoporous carbon. (c,d) AFM height micrographs of PS-*b*-PEO/resols films after (c) spin-coating (as-spun) and (d) vacuum oven heat treatment at 110 °C for 24 h.

We first investigated the ordered morphology of PS-*b*-PEO/resols hybrid bulk monolith samples generated by evaporation-induced self-assembly at 50 °C and thermal curing at 110 °C. The oligomeric resol additive is selectively attracted to the hydrophilic PEO block via hydrogen bonding.^8,10,30^Figure 1a shows the SAXS pattern of the all-organic hybrid monolith (sample i) with a PS-*b*-PEO/resol mass ratio of 7:3, exhibiting strong and intense reflections at angular positions of (*q*/q*)^2^ = 1, 4, 9, 16, and 25, consistent with the lamellar morphology. The principal peak at q* = 0.162 nm^–1^ indicates the *d*-spacing of the lamellar mesostructure as 38.8 nm. To verify the periodic structure, the hybrid monolith was pyrolyzed at 600 °C and characterized by SAXS and TEM. The disappearance of scattering peaks in the SAXS pattern (sample ii in Figure 1a) indicates the bulk mesoporous carbon collapsed into sheet-like structures that was corroborated by TEM (Figure 1b).

**Figure 2:**
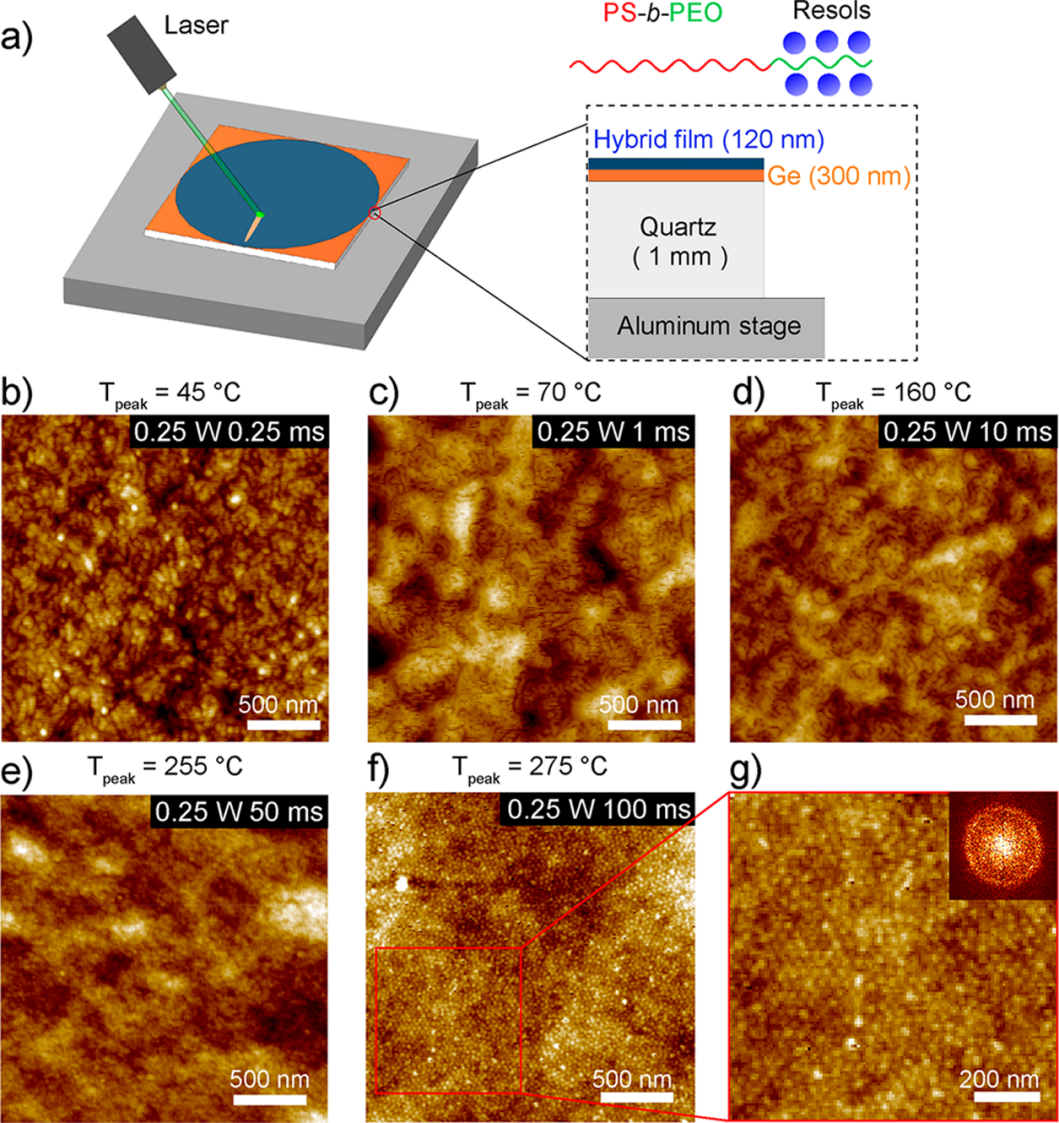
(a) Schematic of LSA of PS-*b*-PEO/resol film on Ge/quartz substrate. (b) AFM height micrographs of PS-*b*-PEO/resol hybrids after LSA at 0.25 W for dwell times of (b) 0.25 ms, (c) 1 ms, (d) 10 ms, (e) 50 ms, and (f,g) 100 ms. The highlighted region in (f) is shown in (g) at a higher magnification. Inset in (g) shows the corresponding FFT profile.

After establishing that the PS-*b*-PEO/resol hybrids have sufficiently high interaction parameters to spontaneously microphase separate into the lamellar lattice under equilibrium, we prepared complementary thin films with the same mass ratio by spin-coating on Ge/quartz substrates (see cross-sectional schematic in Figure 2a). Figure 1c shows the AFM profile of as-formed hybrid film with the kinetically trapped worm-like short cylinder morphology^23^ due to rapid solvent evaporation during spin-coating and interfacial energy effects.^17,28^ We attempted to anneal the hybrid film via equilibrium heat treatment in a vacuum oven at various temperatures from 100 to 150 °C, i.e., above the glass transition temperature of PS (*T*_g_ ∼ 100 °C). However, there were no discernible morphologies on the surface of oven-annealed PS-*b*-PEO/resol films; for example, see the representative AFM image after oven annealing at 110 °C in Figure 1d.

**Figure 3:**
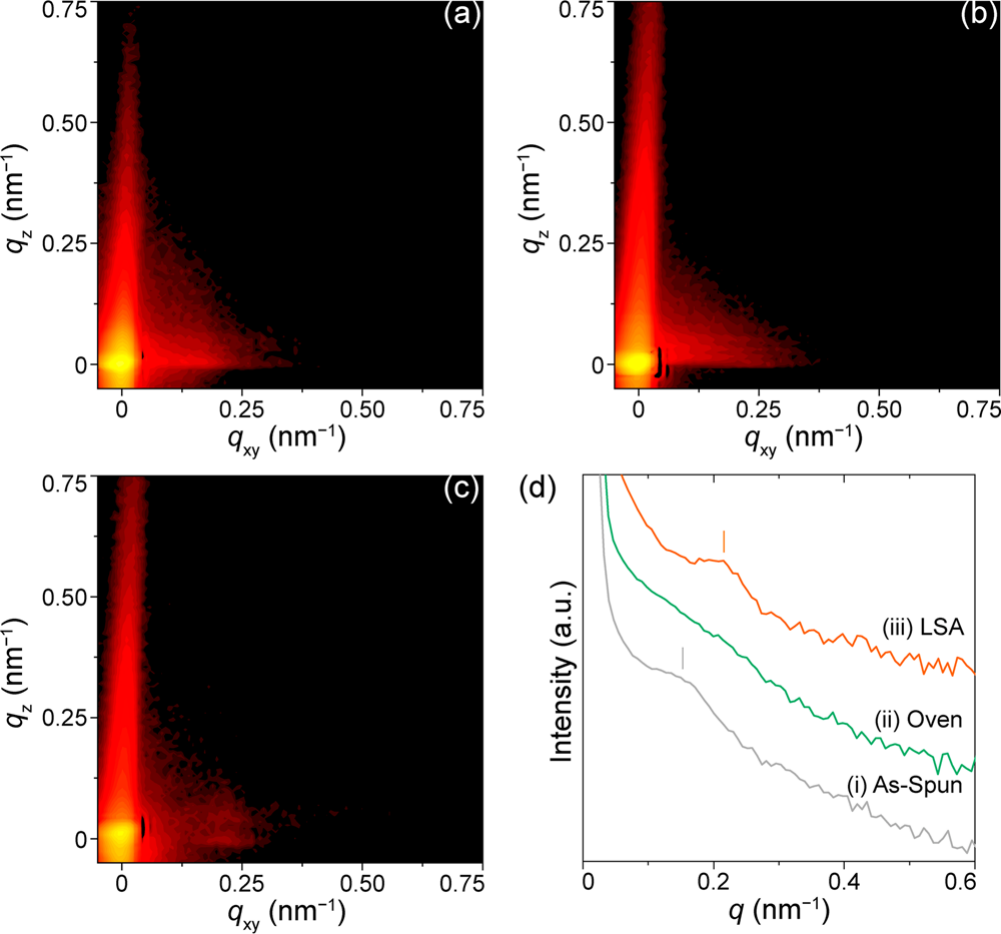
(a–c) GISAXS profiles of PS-*b*-PEO/resol hybrid films after (a) spin-coating (as-spun), (b) vacuum oven annealing at 100 °C for 24 h, and (c) LSA at 0.25 W for 100 ms, measured at incidence angles of 0.18, 0.14, and 0.12°, respectively. (d) Integrated intensity plots of corresponding GISAXS patterns as indicated. Tick marks indicate the angular positions of the principal peaks (q*) of as-spun and laser-annealed films at 0.152 and 0.216 nm^–1^, respectively.

The Ge/quartz substrates were first exposed to ambient air plasma, rendering the surface hydrophilic, followed by hybrid film deposition.^43^ The polar PEO/resol domains prefer to wet the plasma-activated Ge surface, while the lower surface energy PS (40.7 mJ/m^2^) tends to form an upper PS-rich/air interface (note that the surface energy of PEO is 43 mJ/m^2^).^44,45^ We postulate the PS-*b*-PEO/resol films may be displaying two types of ordering behaviors under equilibrium thermal annealing: (1) At elevated oven temperatures (≥100 °C), PS and PEO/resol microdomains gain mobility to rearrange from as-formed disordered structure into the lamellar lattice in which the microdomains are oriented parallel to the substrate (in-plane direction).^21^ (2) Alternatively, higher temperatures could promote thermal polymerization of resol additive into higher molar mass resin, lowering the overall film mobility to reorganize into an ordered morphology. Figure 3a,b shows the grazing incidence small-angle X-ray scattering (GISAXS) measurements of as-formed and oven-annealed hybrid film samples, respectively. However, the absence of pronounced GISAXS reflections suggests that the resultant hybrid films were disordered even after vacuum oven thermal annealing, consistent with the AFM data analysis.^11,28^

### LSA of PS-*b*-PEO/Resols Films at 0.25 W

LSA enables ultrafast heating of polymer films at high temperatures (>800 °C) for dwell times as short as 10^–5^ s, followed by rapid quenching at fast rates (up to 10^7^ K/s), thereby providing thermodynamic and kinetic control of BCP morphological developments via tuning the diffusivity and reaction rate of respective polymer blocks.^20,38^Figure 2a shows the schematic of LSA of PS-*b*-PEO/resol hybrid films scanned by a 532 nm continuous-wave laser with a focused line beam profile fwhm of 100 μm by 400 μm. Heating dwell time is defined by beam fwhm divided by the scan velocity. The Ge overlayer absorbs the laser photons to convert into thermal energy to anneal the hybrid films, followed by cooling via thermal conduction into the quartz substrate.^18,38,40^ Thus, laser power and heating dwell time govern both the LSA heating/cooling rates and durations as well as peak laser annealing temperatures (*T*_peak_) in the center of the laser scan line.^38,40,41^ The *T*_peak_ values derived from FEM simulations and absolute temperature calibrations using the PS homopolymer standard (Figure S2) are summarized in Table 1.^18,40,41^Figure 2b–g shows the microstructure evolution of PS-*b*-PEO/resol films after LSA for heating dwell times of 0.25 to 100 ms, all at the laser power of 0.25 W, corresponding to *T*_peak_ of 45 to 275 °C.

**Table 1:** LSA Characteristics of PS-*b*-PEO/Resol Hybrid Films

power (W)	dwell time (ms)	*T*_peak_ (°C)	*t*_order,1_ (ms)	*t*_mixing_ (ms)	*t*_order,2_ (ms)	heating rate (K/s)	quench rate (K/s)	morphology
0.25	0.25	45				10^4^	10^4^	worm-like
	1	70				10^4^	10^3^	mixed cylinders
	10	160	55			10^3^	10^3^	mixed cylinders
	50	255	83	259	257	10^2^	10^2^	random spheres
	70	265	136	393	328	10^2^	10^2^	random spheres
	90	270	195	523	396	10^2^	10^2^	random spheres
	100	275	234	658	449	10^2^	10^2^	bcc spheres
0.5	1	115	2.2			10^4^	10^4^	mixed cylinders
	10	295	8.5	65	118	10^3^	10^3^	disordered
	100	525	312	1469	621	10^2^	10^2^	disordered
1	1	205	0.8	2.7	3.9	10^4^	10^4^	disordered

Although the PS-*b*-PEO/resol film mostly resembles a worm-shaped morphology in the as-formed film (Figure 1c) for the shortest LSA dwell time of 0.25 ms corresponding to *T*_peak_ of 45 °C, we observed small clusters of short-ranged aligned cylinders distributed throughout the AFM profile (Figure 2b), suggesting the microdomains gained some degree of mobility to self-order into a lower free energy state. As heating dwell time increased to 1 and 10 ms, AFM images in Figure 2c,d show that LSA induced a new microstructure comprising a mixture of vertically standing and horizontally lying cylinders. This could be explained by a further increase in chain mobility of PS and PEO/resol domains to rearrange at higher *T*_peak_ values of 70 and 160 °C for 1 and 10 ms dwell times, respectively. Figure 2e shows an impending phase transition at 255 °C for 50 ms, in which the cylindrical morphology disappeared, replaced by a weakly segregating sphere-shaped microstructure. Most interestingly, the AFM profile in Figure 2f shows that LSA at 275 °C for 100 ms resulted in an ordered in-plane sphere morphology with some degrees of hexagonal symmetry distortion, resembling the (110) plane of the body-centered cubic (bcc) structure.^46^ The equivalent AFM image in phase mode displayed in Figure S4a suggests that the spheres were made up of softer PEO/resol domains in the PS matrix. Fast Fourier transform analysis gave a homogeneous in-plane lattice spacing of ∼28.6 nm (Figure S4b), smaller than the *d*-spacing of the bulk equilibrium lamellar lattice. Importantly, the GISAXS profile of the laser-annealed film in Figure 3c exhibits an intense principal peak at q* = 0.216 nm^–1^. This corresponds to an in-plane *d*-spacing of 29.1 nm that is almost identical to that measured in AFM, thereby confirming improved microphase segregation and structure order.^20,23^ It is noted that the absence of higher order reflections may be due to the smaller focused X-ray beam size to improve spatial resolution but also possibly resulting in divergence and reduced flux of the incident X-rays.^20^ We postulate the PS and PEO/resol domains gained maximum chain mobility at *T*_peak_ of 275 °C for a 100 ms dwell time and self-organized during the laser-induced heating and subsequent cooling durations, albeit with the competing thermopolymerization kinetics of resols (vide infra).

### LSA of PS-*b*-PEO/Resols Films at 0.5 W

**Figure 4:**
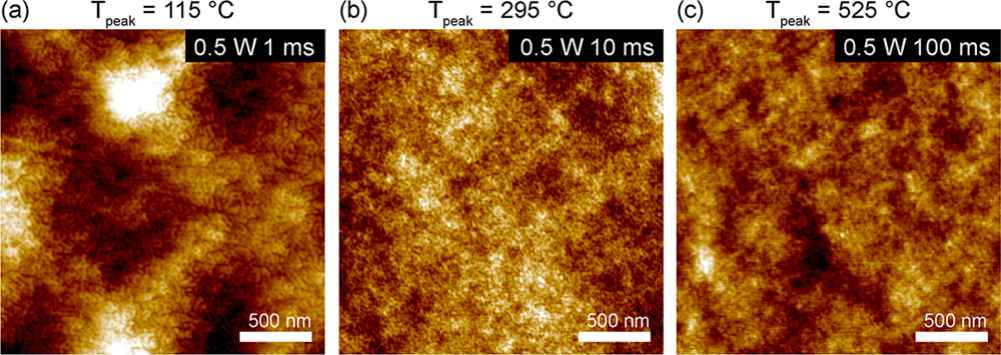
AFM height micrographs of PS-*b*-PEO/resol hybrids after LSA at 0.5 W for dwell times of (a) 1 ms, (b) 10 ms, and (c) 100 ms.

To establish the processing window for LSA-induced ordering, PS-*b*-PEO/resols films were irradiated at 0.5 W and examined by AFM. For the dwell of 1 ms, Figure 4a shows a better resolved morphology of perpendicular and parallel cylinders attributed to higher chain mobility at 115 °C to form a stable microstructure. A major difference in this set of experiments, however, is that the microstructures of PS-*b*-PEO/resol became increasingly disordered with longer heating dwell times, as displayed in Figure 4b,c. We speculate the rapid increase in *T*_peak_ to 295 and 525 °C for dwell times of 10 and 100 ms, respectively, may have amplified thermopolymerization of resols into a larger molar mass resin, thereby reducing the overall hybrid film mobility and ordering kinetics (vide infra).

### LSA-Induced Phase Separation and Ordering Kinetics of PS-*b*-PEO/Resol Films

The PS-*b*-PEO/resol hybrid composite is unique as it begins as a “soft” combination of structure-directing di-BCP mixed with oligomeric resol additives selectively attracted to the PEO block by hydrogen bonding.^10,34^ Ordered hybrids and mesoporous resin/carbon microstructures could be obtained by solvent annealing^30^ and oven thermal annealing^35^ techniques that require processing durations ranging from a few minutes to several hours. For example, Bein and co-workers employed in situ GISAXS to probe the thermal annealing behaviors of Pluronic F127/resol films at 100 °C.^35^ Formation of the hexagonal cylindrical microstructure was completed around the 40 min time point. They proposed that structure formation in BCP/resols film occurred during the thermopolymerization of resols together with higher polymer diffusion kinetics at elevated temperatures.^35^ While structure formation rates were accelerated with faster ramp rates to higher annealing temperatures, no alternative microstructure was observed.^35^

**Figure 5:**
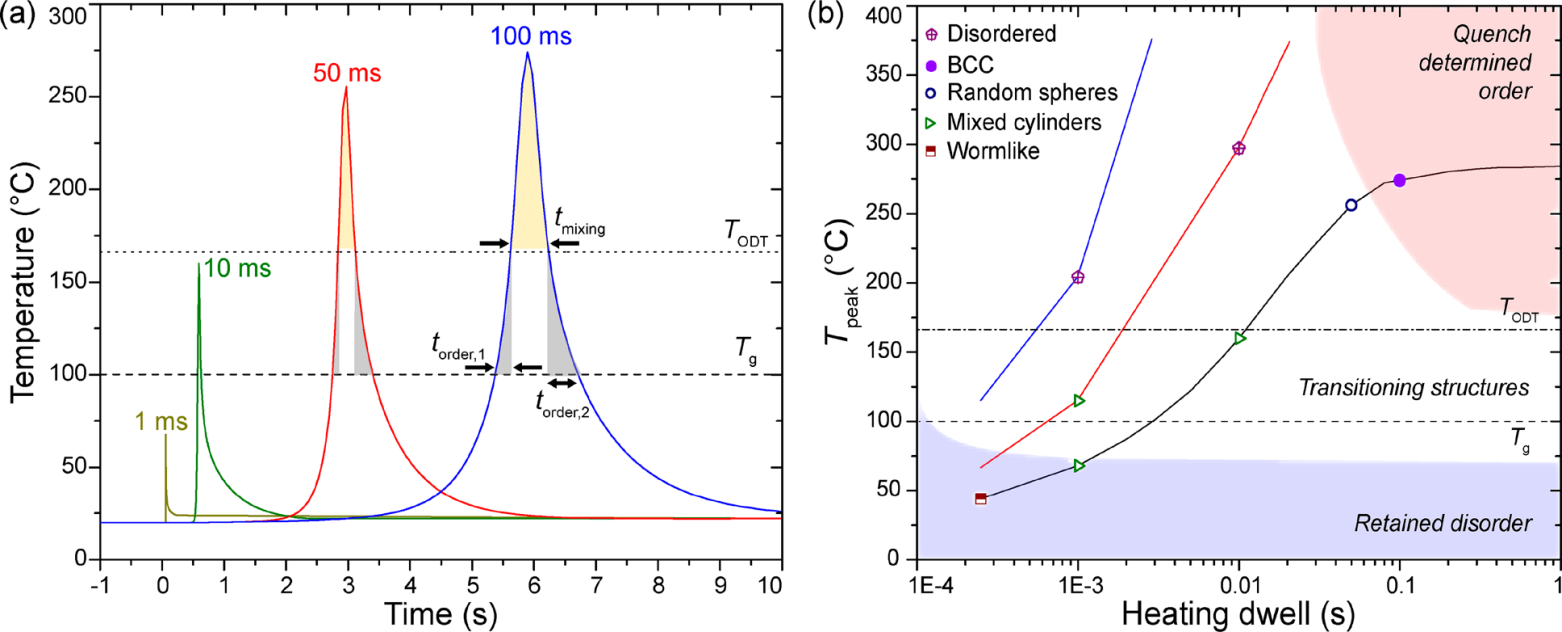
(a) Simulated peak annealing temperature profiles with a laser power of 0.25 W for dwell times of 1, 10, 50, and 100 ms, as indicated. The plots are offset horizontally for clarity. (b) Morphology map of laser-heated PS-*b*-PEO/resol hybrid films as a function of peak LSA temperature and heating dwell time. The colored regions and lines are guides for the eye only.

In another studies, Jacobs and co-workers^20,23^ identified two kinetic regimes in the LSA of neat BCP films as a function of *T*_peak_ and dwell times (e.g., see Figure 5a): (1) ordering regime for times spent with laser annealing temperatures above *T*_g_ but below the order–disorder transition temperature *T*_ODT_ (*T*_g_ < *T* < *T*_ODT_), furthering microphase separation and long-range ordering; (2) mixing regime for very short times with temperatures above *T*_ODT_ (*T* > *T*_ODT_) that promotes defect elimination but also disorder. They proposed the ideal LSA process window toward defect-free, periodic BCP microstructures with long-range order should have annealing temperatures above *T*_ODT_ for very brief durations (short mixing regime) to efface any existing defects and then to hold the temperatures above *T*_g_ but below *T*_ODT_ (*T*_g_ < *T* < *T*_ODT_) for sufficiently long times to enable high polymer diffusivities and to initiate the nucleation and growth of ordered microstructures with long-range correlations.^20,23^

To elucidate the LSA-induced self-assembly mechanism, we plotted the peak annealing temperature profiles of PS-*b*-PEO/resol films heated at 0.25 W for dwell times of 1, 10, 50, and 100 ms displayed in Figure 5a. The critical temperatures, defined by *T*_g_ of PS (∼100 °C) and *T*_ODT_ (∼166 °C) obtained from differential scanning calorimetry measurements of the bulk hybrid with lamellar microstructure (Figure S5),^47^ enabled estimation of the LSA ordering (*T*_g_ < *T* < *T*_ODT_) and mixing (*T* > *T*_ODT_) durations summarized in Table 1.

For the shortest dwell time of 0.25 ms after laser irradiation at 0.25 W, the hybrid film heated at *T*_peak_ of 45 °C remained kinetically limited and thus retained the short worm-like cylinder microstructure. Interestingly, even though the *T*_peak_ of 70 °C for 1 ms dwell time is lower than the critical *T*_g_ limit (100 °C), the hybrid film rearranged into the mixed morphology of vertical perpendicular and horizontal parallel cylinders (Figure 2c). This could be attributed to higher diffusivities of the minority PEO block (*T*_g_ of PEO ≈ −56 °C) and the oligomeric resol additives. LSA at a 10 ms dwell time provided increased mobility to the PS domains as *T*_peak_ of 160 °C crossed the lower critical *T*_g_ limit, thus entering the ordering regime (green curve in Figure 5a). This is consistent with the better resolved mixed cylindrical microstructure observed in AFM (Figure 2d).

For LSA dwell times of 50 and 100 ms, the PS-*b*-PEO/resol films underwent disordering (mixing regime) as *T*_peak_ values exceeded *T*_ODT_ to reach 255 and 275 °C, respectively (red and blue curves in Figure 5a). Hence, we expect the initial phase segregation and equilibrium defects in the films to be first expunged and then transit to an alternative morphology during quench (ordering regime).^20,23^ This was confirmed in the AFM profiles exhibiting spherical microstructures after LSA. In particular, the PS-*b*-PEO/resol film sample heated for the longer 100 ms dwell time displayed the most long-range-ordered in-plane bcc spherical morphology (compare Figure 2e,f). This was affirmed in the GISAXS profile exhibiting an intense diffraction peak (Figure 3c).

Given the similar *T*_peak_ values (255 versus 275 °C), to first order, the diffusivities of microdomains would not be very different for LSA dwell times of 50 and 100 ms after irradiation at 0.25 W. Instead, it is essential to provide enough time for the complete removal of kinetically trapped segregated state and defects (*t*_mixing_) but also short enough to inhibit formation of new defects during nucleation and growth of long-range-ordered microstructure during quench (*t*_order,2_). LSA of PS-*b*-PEO/resol films at 0.25 W for a 100 ms dwell time provided the most optimal laser annealing conditions; that is, the durations of mixing (*t*_mixing_) and ordering during quench (*t*_order,2_) are larger than that of the 50 ms LSA dwell time by factors of 2.5 and 1.7, respectively. Indeed, in control experiments, we observed the laser-annealed PS-*b*-PEO/resol films exhibiting progressively well-resolved spherical microstructures as LSA dwell times increased from 50 to 90 ms at 0.25 W, corresponding to longer *t*_mixing_ and *t*_order_ durations for improved long-range ordering (Figure S6a–c). The morphology difference between *non-equilibrium* LSA-induced thin film bcc structure and *equilibrium* bulk lamellar structure may be attributed to the difference in annealing histories, surface and interfacial energy contributions, as well as the increase in segregation strength (χ*N*, product of Flory–Huggins interaction parameter χ and total number of repeating units *N*) due to the thermopolymerization of resol additives.^17,35^

We further note that *T*_peak_ and laser heating rates also greatly impact the thermopolymerization kinetics of resol additives and thereby resultant PS-*b*-PEO/resol microphase segregation behaviors, as demonstrated in LSA experiments at 0.5 W. *T*_peak_ was increased to 115 °C (>*T*_g_) after irradiation at 0.5 W for 1 ms, facilitating formation of mixed cylindrical morphology (Figure 4a) with higher polymer diffusion kinetics. It came as a surprise, however, that LSA with longer dwell times resulted in disordered films (Figure 4b,c). We posit that LSA at 0.5 W for 10 ms resulted in higher *T*_peak_ (295 °C) and heating rates (10^3^ K/s) that augmented resol thermopolymerization into higher molar mass resin, reducing the overall film mobility and ordering kinetics. LSA of 100 ms at 525 °C resulted in even higher degrees of resol thermopolymerization and total immobilization of polymer domains. It should be mentioned that LSA of PS-*b*-PEO/resol films at higher laser powers (e.g., 1 W) yielded disordered microstructures and film damage (Figure S6d).^20^ Finally, we summarized our observations in a laser annealing temperature–dwell time–transformation morphology map presented in Figure 5b to describe the LSA-induced self-assembly behaviors.^20^

## Conclusion

In summary, we conducted millisecond laser spike annealing of all-organic PS-*b*-PEO/resol hybrid films and observed disorder–order morphology transitions as a function of laser powers (peak annealing temperatures) and heating dwell times for the first time. In particular, we discovered that laser annealing at higher temperatures far in excess of *T*_g_ and *T*_ODT_ for millisecond time scales provided access to ordered BCP morphologies different from the bulk equilibrium structure. We showed that the degree of ordering in hybrid films improved as laser annealing temperatures increased (200–300 °C) at moderate rates (∼10^2^ K/s) with longer dwell times (10–100 ms), thereby providing control to tune diffusivities of polymer domains, thermopolymerization kinetics of resols, and the resultant film mixing and ordering behaviors. For this particular PS-*b*-PEO/resol film combination, we obtained an ordered in-plane spherical bcc morphology after laser irradiation at 0.25 W for 100 ms (*T*_peak_ = 275 °C). Our results are summarized into a laser annealing temperature–dwell time–transformation morphology map that could serve as a guide to access new chemistries and explore alternative ordered morphologies of other BCP/resol combinations and novel composite systems.

## Supporting Information Available

Additional experimental descriptions and characterization of PS-*b*-PEO/resol hybrid and PS hompolymer films by AFM, DSC, TGA, profilometry and optical microscopy (PDF)
