# Supplementary material for: Transient Laser-Annealing-Induced Mesophase Transitions of Block Copolymer–Resol Thin Films
Source: ACS Polym Au. 2021 Dec 13;2(1):42–9. doi: 10.1021/acspolymersau.1c00040 (PMC9954231; doi:10.1021/acspolymersau.1c00040)
Supplement: Supplementary file 1 — lg1c00040_si_001.pdf [file lg1c00040_si_001.pdf]

Supporting Information for

Transient Laser Annealing Induced Mesophase Transitions of Block Copolymer–Resols Thin Films

Wei Han Tu,[†,⊥] *Geok Leng Seah,*[†,⊥] *Yun Li,*[†] *Xinghui Wang,*[‡] *Kwan W. Tan*[†,*]

[†]School of Materials Science and Engineering, Nanyang Technological University, Singapore 639798, Singapore

[‡]College of Physics and Information Engineering, Institute of Micro-Nano Devices and Solar Cells, Fuzhou University, Fujian, China

[⊥]These authors contributed equally to the work.

[*]Corresponding Author. Email: kwtan@ntu.edu.sg

This PDF file includes:

Additional Experimental Section. S2
Figure S1. Schematic of Ge/quartz substrate for FEM simulation. S4
Figure S2. Absolute temperature calibration using PS homopolymer. S5
Figure S3. Plots of simulated peak temperature versus laser power. S6
Figure S4. AFM phase profile of laser-annealed hybrid film. S7
Figure S5. DSC analysis of bulk PS-*b*-PEO/resols monoliths. S8
Figure S6. Additional AFM profiles of laser-annealed hybrid films. S9
Figure S7. Additional simulated peak annealing temperature profiles. S10

ADDITIONAL EXPERIMENTAL SECTION

Synthesis of macroinitiator PEO_{140}-Br. 10 g of poly(ethylene glycol) methyl ether (PEO-OH, average M_n 5000 g/mol) was dissolved in 30 ml of dichloromethane (DCM) in a Schlenk flask. 3 cycles of freeze-pump-thaw were performed, followed by addition of 2.0 ml of triethylamine. After gentle N_2 purging for 30 min, 1.5 ml of 2-bromoisobutyryl bromide was slowly added while stirring at 0 °C (ice bath) for 3 h. The reaction mixture was then left at room temperature for 20 h under stirring. The resulting mixture was washed using equal volumes of deionized water (DI-H_2O) and DCM in a separating funnel to remove the insoluble by-products – triethylamine salt and unreacted PEO-OH. The DI-H_2O/DCM washing step was repeated two more times. Finally, the polymer solution in DCM was precipitated in excess diethyl ether. The final PEO-Br was dried at room temperature for 24 h under vacuum. Our calculated yield was approximately 75.8%.

ATRP synthesis of PEO-*b*-PS. 1.0 g of PEO_{140}-Br was dissolved in 5.15 g of styrene monomer and 3 ml of anhydrous anisole in a three-neck reaction flask. 0.0042 g of $CuBr_2$ solid was quickly added. The reaction flask was sealed with rubber septum stoppers and purged with N_2 for 30 min, followed by 4 cycles of freeze-pump-thaw to remove moisture and oxygen. In a separate vial, 0.0242 g of CuBr was dissolved in 1 ml of anhydrous anisole and mixed with 0.035 g of PMDETA. The catalyst solution was stirred for 30 min to ensure complete dissolution of CuBr. The catalyst solution was then injected into the reaction flask under N_2 followed by a cycle of freeze-pump-thaw. The rubber septum sealed reaction flask was placed in an oil bath at 110 °C for 24 h under stirring. The reaction was terminated by opening the seal and exposing the catalyst to air. ~40 ml of tetrahydrofuran was added into the flask to reduce the mixture viscosity. The mixture was then passed through a neutral alumina column and precipitated in excess methanol. The PS-*b*-PEO flakes were fully dried on a vacuum line for another 24 h.

Finite element modeling simulations. The 2D finite element modeling (FEM) simulation model is a Ge/quartz substrate with a 25-mm-long by 300-nm-thick Ge overlayer on a 25-mm-long by 1-mm-thick quartz substrate that is placed on a 100-mm-long by 10-mm-thick aluminum block (dynamic linear stage). The 532 nm laser heat source, Q (eqs S1 and S2), was modelled as a Gaussian line with beam profile r_x and r_y of 340 μm and 85 μm, respectively, where $r =$

$(1.699 \times FWHM)/2$. It is reasonable to assume the Ge/quartz substrate is thermally isotropic and the Ge germanium overlayer absorbs the laser photons to heat the polymer films. All surfaces were subjected to surface-to-ambient radiation using eq S3. The simulated peak temperature T of Ge/quartz substrate was computed using eq S4. All materials properties are summarized in Table S1 and a schematic of the FEM mesh model is shown in Figure S1. The pre-defined element size has the highest resolution and total number of nodes in the mesh model is 456407 (see Figure S1).

$$Q = (1 - R)\left(\frac{2P\alpha}{\pi r_x r_z}\right) exp\left(\frac{-(r_{focus})^2}{2}\right) exp(-\alpha y) \quad \text{(S1)}$$

$$r_{focus} = \frac{4\left(x - \frac{r_x t}{t_{dwell}}\right)^2}{r_x r_z} \quad \text{(S2)}$$

$$n \cdot (k\nabla T) = \varepsilon\sigma(T_0^4 - T^4) \quad \text{(S3)}$$

$$Q = \rho C_p \frac{\partial T}{\partial t} - \nabla(k\nabla T) \quad \text{(S4)}$$

Table S1. Constants used in the FEM simulation.

Property	Ge	Quartz	Al	Laser
R, reflective index,	0.515			
α (cm^{-1}), absorption coefficient	564000			
r_x (µm), beam width (FWHM)				50
r_z (µm), beam length (FWHM)				200
P (W), laser power				0.25–1
t_{dwell} (ms), laser annealing dwell				100
T_0 (K), ambient temperature	293.15	293.15	293.15	
ρ (kg m^{-3}), density	5323	2210	2700	
k (W K m^{-1}), thermal conductivity	58	1.4	238	
C_p (J K kg^{-1}), specific heat capacity	310	730	900	
ε, surface emissivity	0.42	0.7	0.057	

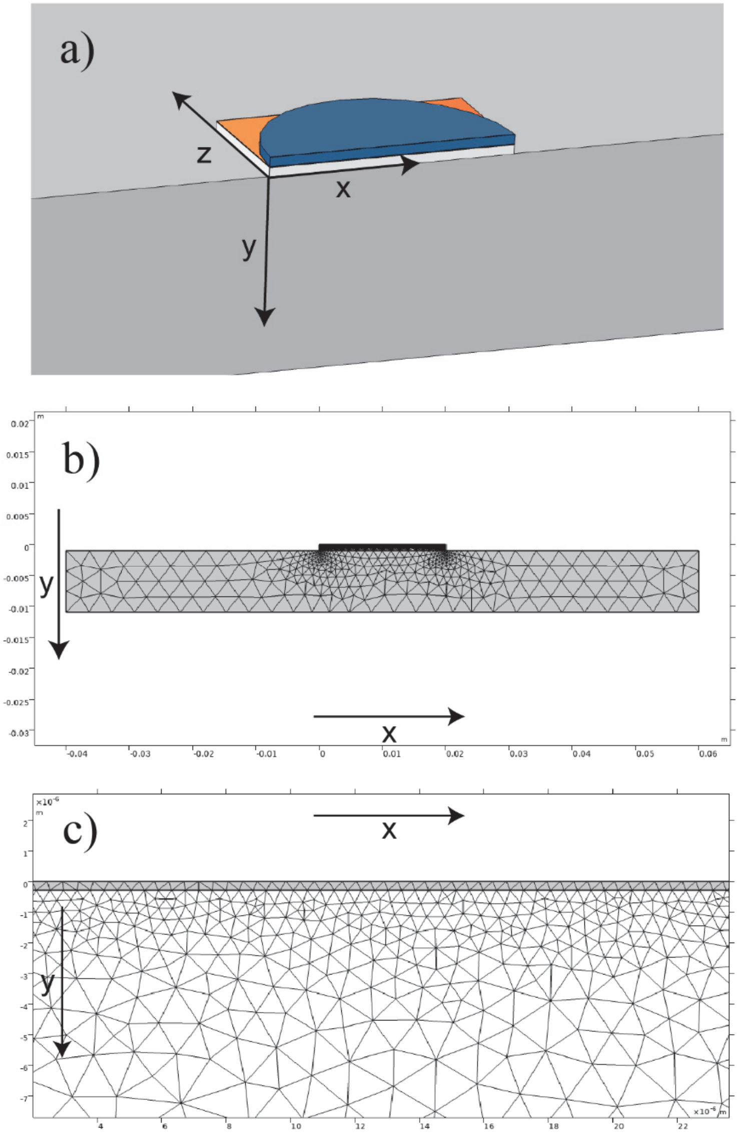

Figure S1. Schematic of Ge/quartz substrate for FEM simulation. (b, c) Ge/quartz mesh model under different magnifications.

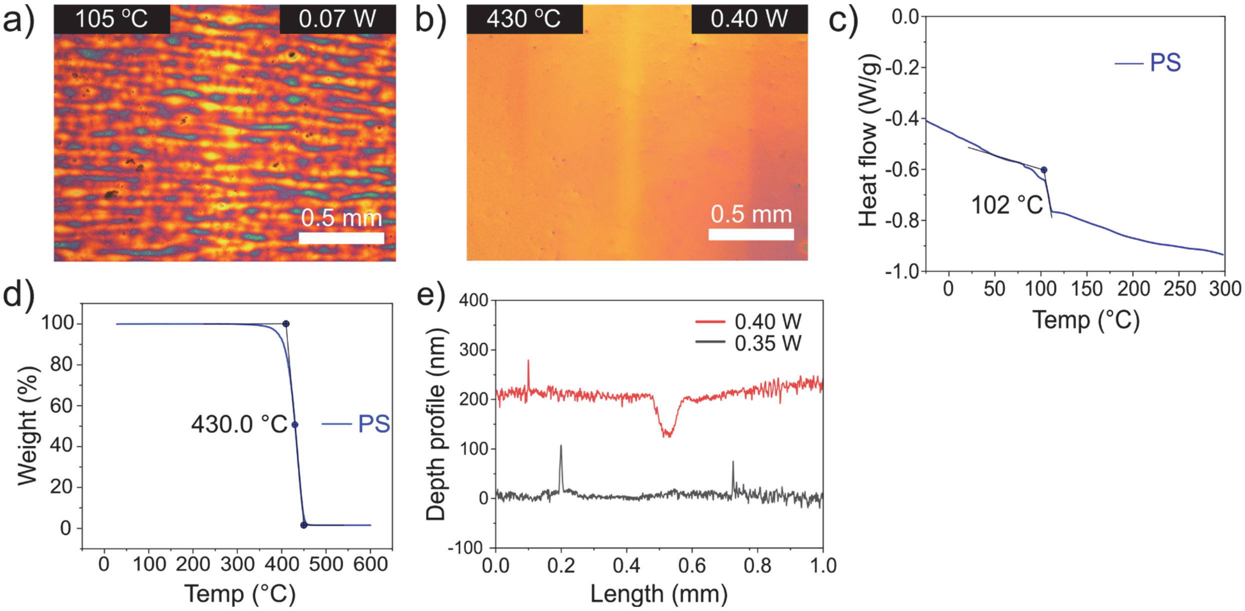

Figure S2. (a, b) Optical images of thin films of PS on Ge/quartz substrates heated by a single laser irradiation of 100 ms dwell at laser powers of 0.05 to 0.5 W. The visible laser scanned lines indicate laser-induced glass transition and decomposition behaviors. (c, d) DSC and TGA data of PS samples show the glass transition and decomposition temperatures at ~102 and ~430 °C, respectively. (e) Profilometry data of PS thin films after laser annealing at 0.35 and 0.4 W for 100 ms dwell. The plots are offset vertically for clarity. There was no observable change in PS film thickness after laser heating at 0.35 W (dark grey curve). In contrast, the film thickness reduction of PS film after laser annealing at 0.4 W (red curve) indicates laser-induced thermal decomposition, corresponding to the decomposition temperature of 430 °C obtained by TGA.

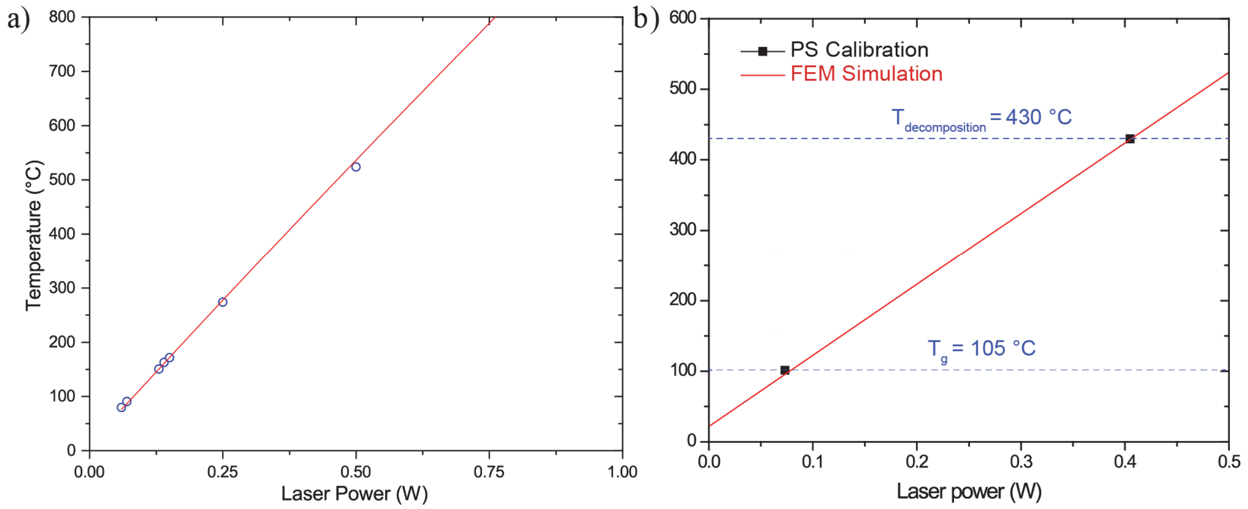

Figure S3. (a, b) Plots of FEM simulated peak temperature versus laser power. The black data points in (b) denote the absolute temperature calibration based on laser-induced glass transition and decomposition transformations of PS thin films at 105 and 430 °C, respectively, showing close agreement with the simulated peak temperature–laser power curve.

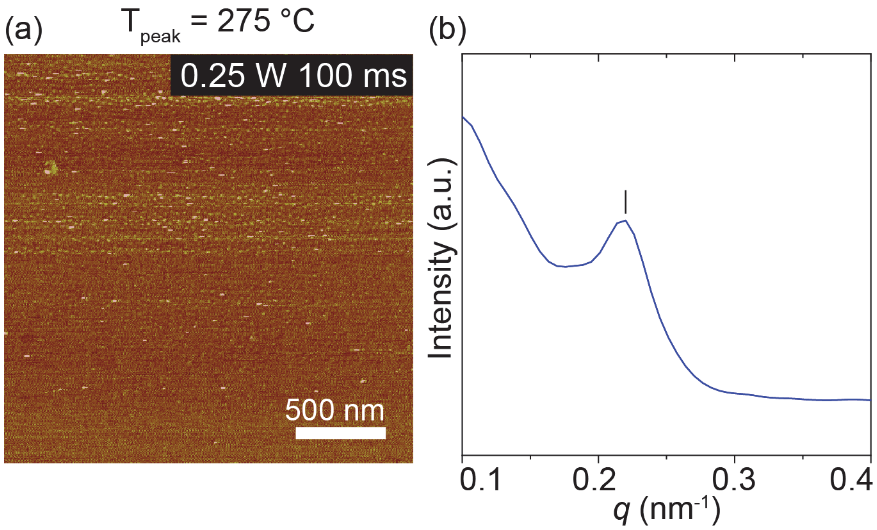

Figure S4. (a) AFM phase micrograph and (b) FFT analysis of PS-*b*-PEO/resols film after LSA at 0.25 W for 100 ms. The peak of the azimuthally integrated FFT curve corresponds to an in-plane lattice spacing of $2\pi/q \approx 28.6$ nm.

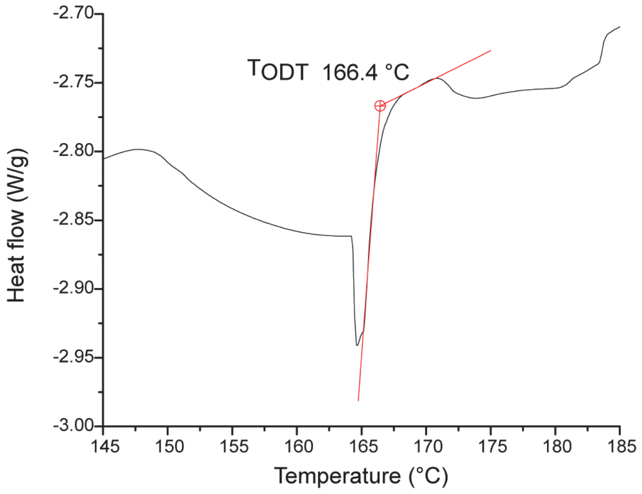

Figure S5. DSC heating curve of PS-*b*-PEO/resols bulk hybrid monolith with lamellar mesostructure.

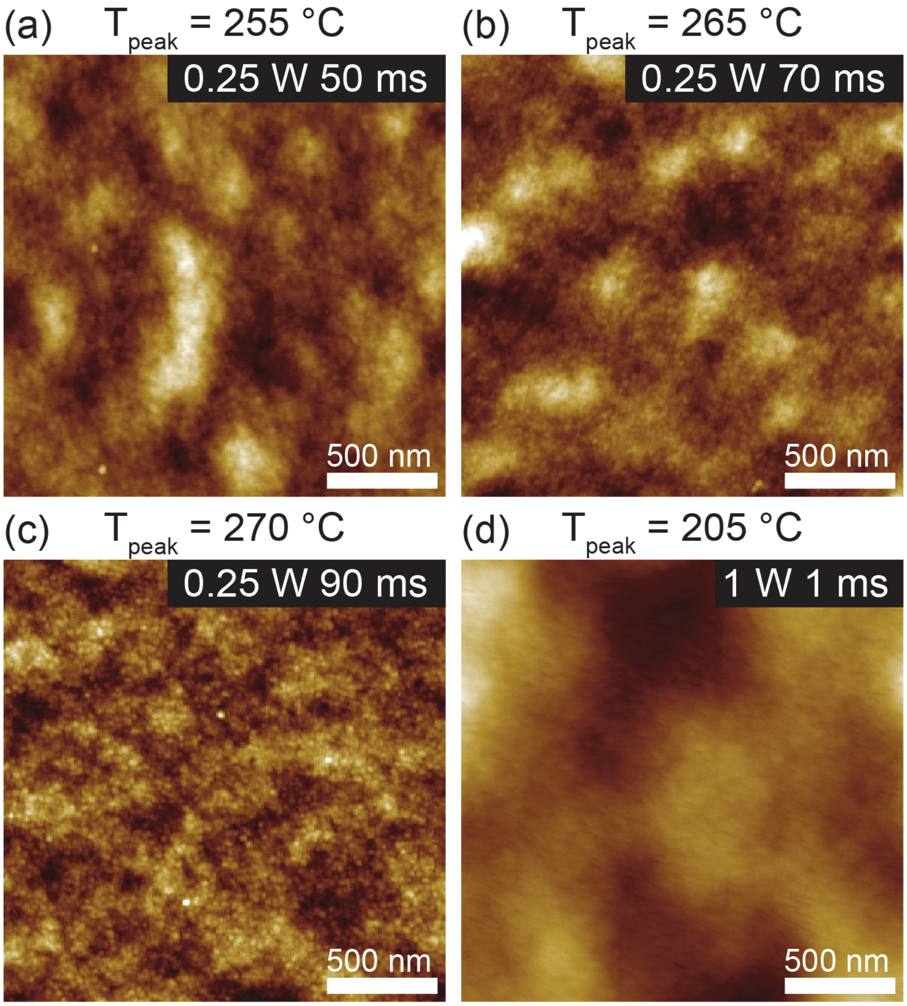

Figure S6. (a–d) AFM height profiles of PS-*b*-PEO/resols hybrid films heated by a single laser irradiation of 0.25 W and 1 W for different dwells as indicated. The sphere-type morphology becomes increasingly evident with longer heating dwells, see panels (a) to (c). The AFM height profile in panel (d) is indiscernible, suggesting negligible microphase segregation despite reaching the peak temperature of 205 °C (>T_{ODT} = 166 °C).

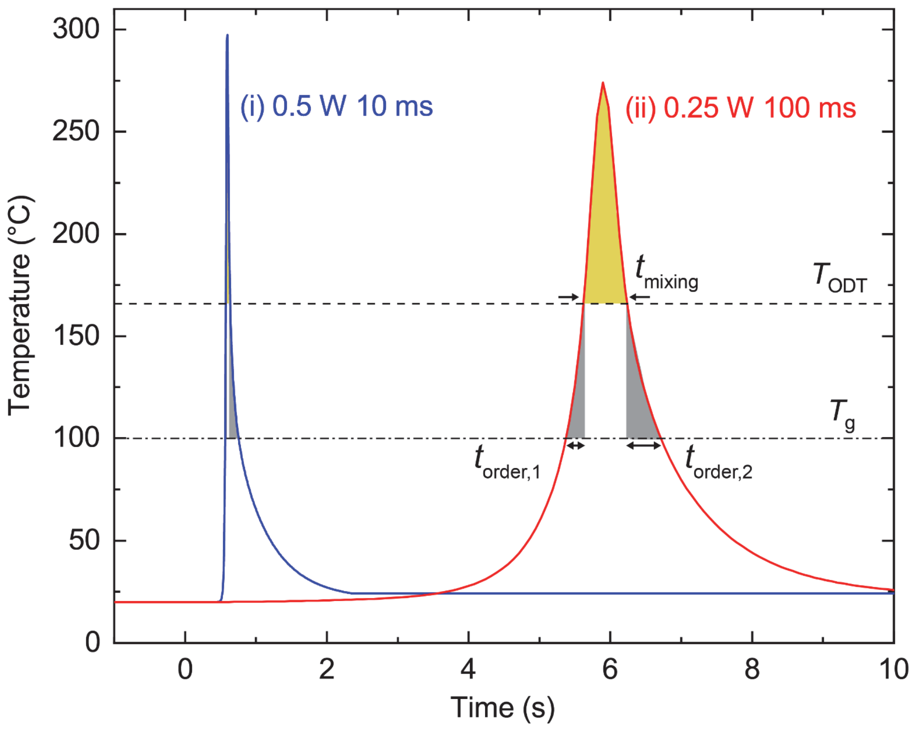

Figure S7. Peak temperature profiles for laser annealing parameters of (i) laser power 0.5 W for 10 ms and (ii) laser power 0.25 W for 100 ms. The plots are offset horizontally for clarity. Despite reaching similar peak temperatures of 275 to 300 °C, the mixing and ordering durations are highly dependent of the respective LSA dwell.
